# Low-Frequency Ultrasound Coupled with High-Pressure Technologies: Impact of Hybridized Techniques on the Recovery of Phytochemical Compounds

**DOI:** 10.3390/molecules26175117

**Published:** 2021-08-24

**Authors:** Giovani Leone Zabot, Juliane Viganó, Eric Keven Silva

**Affiliations:** 1 Laboratory of Agroindustrial Processes Engineering (LAPE), Federal University of Santa Maria (UFSM), Cachoeira do Sul 96508-010, Brazil; giovani.zabot@ufsm.br; 2 School of Applied Sciences (FCA), University of Campinas (UNICAMP), Limeira 13484-350, Brazil; juvigano@unicamp.br; 3 School of Food Engineering (FEA), University of Campinas (UNICAMP), Campinas 13083-862, Brazil

**Keywords:** supercritical CO_2_, pressurized liquids, gas-expanded liquids, acoustic cavitation

## Abstract

The coupling of innovative technologies has emerged as a smart alternative for the process intensification of bioactive compound extraction from plant matrices. In this regard, the development of hybridized techniques based on the low-frequency and high-power ultrasound and high-pressure technologies, such as supercritical fluid extraction, pressurized liquids extraction, and gas-expanded liquids extraction, can enhance the recovery yields of phytochemicals due to their different action mechanisms. Therefore, this paper reviewed and discussed the current scenario in this field where ultrasound-related technologies are coupled with high-pressure techniques. The main findings, gaps, challenges, advances in knowledge, innovations, and future perspectives were highlighted.

## 1. Introduction

The development of innovative processes based on low-frequency and high-power ultrasound technology has grown in the last two decades due to its large versatility and application in several industrial sectors. Low-frequency ultrasound has been used to enhance from technological properties of polymers [1,2] to manufacturing processes of foods and beverages [3,4,5], alloys, and composite materials [6,7], in addition to its application in many research fields, such as tissue engineering [8,9], synthesis of chemicals [10], design of novel encapsulating systems [11,12], extraction of phytochemical compounds [13], and many others.

**Figure 1:**
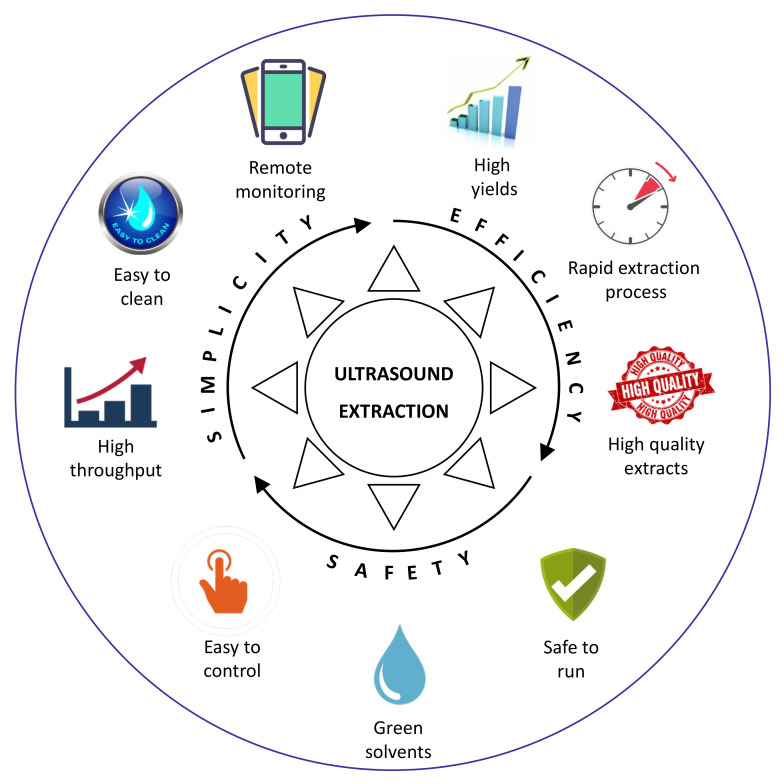
Advantages of ultrasound technology for the extraction of target compounds.

Regarding the ultrasound-assisted extraction processes, the ultrasound performance as a process improver is associated with its action mechanism, whose principle is the acoustic cavitation phenomenon. Acoustic fields promote the formation and subsequent collapse of microbubbles, converting acoustic energy into thermal and mechanical energy. Thereby, the acoustic cavitation enhances the mass transfer convective coefficients due to increased temperature and turbulence, resulting in more efficient extraction processes [13,14,15]. A scheme that shows why ultrasound extraction is a promising technology is presented in Figure 1.

Indeed, the use of low-frequency ultrasound has been widely studied as a means of intensifying the extraction process while preserving the functionality and biological activity of plant extracts [13]. In this regard, the coupling of low-frequency ultrasound to other clean emerging technologies to get synergetic effects is an interesting strategy for recovering bioactive compounds from plant matrices. Furthermore, hybridized techniques based on sonoprocessing and high-pressure technologies, such as supercritical fluid extraction, pressurized liquids extraction, and gas-expanded liquids extraction, have emerged as promising alternatives to approach sustainability, since this is a big worldwide goal. Therefore, this review presents and discusses in the following sections the current scenario in this field where ultrasound-related technologies are used, highlighting the main findings, gaps, challenges, advances in knowledge, and future perspectives. This review was prepared based on searches done in the scientific and technical databases. Technical reports, scientific papers, and patents, among others, were consulted and discussed. The searches were done using the main strings such as “ultrasound” and/or “extraction” and/or “supercritical” and/or “pressurized” and/or “high-pressure technology”. The main relevant data were considered, where the authors were properly referenced. The revision was focused on recent outcomes, especially from 2015–2020. However, classical and important former reports were also considered. The authors’ experience was inserted in this revision to give a particular viewpoint and future outlooks in this promising area. This is certainly not the first review that evaluates and extolls the virtues of ultrasound-based extraction of target compounds from renewable biomasses. Despite that, it makes a relevant contribution to food-related and chemical-related areas specifically on the challenges and future perspectives of using ultrasound technology coupled with high-pressure technologies.

## 2. Fundamentals and Mechanisms

Obtaining bioactive compounds from the matrix of solid raw materials involves a crucial step—extraction. Extraction techniques can be classified into those using solvents or mechanical expelling to accomplish the process. Bioactive compounds are, in general, minor compounds in the raw materials and require solvent extractions to be recovered. Bioactive extract production with simultaneous high yield and high extract concentration in a shorter processing time is challenging. In addition to these aspects, the extraction technique is expected to meet other issues such as environmental regulatory demands, for example. Given these questions, several extraction techniques have emerged in the past two decades. This section will discuss the fundamentals of some of them, namely low-frequency ultrasound, high-pressure, and their hybridization.

### 2.1. Low-Frequency Ultrasound Technology

Ultrasound comprises mechanical waves that need an elastic medium to propagate and has a broad spectrum of applications. Classification of ultrasound technology can be based on the frequency and energy level at which ultrasound propagates through the medium, characterized by sound power (W), sound intensity (W/m^2^) or sound energy density (W/m^3^). The ultrasound applications are defined into two groups: low-intensity ultrasound that works at high frequency (100 kHz–1 MHz) and low power (typically < 1 W/cm^2^); and high-intensity ultrasound that operates at low frequency (16–100 kHz) and high power (typically 10–1000 W/cm^2^). High-frequency ultrasound is mainly applied in non-destructive applications, while low-frequency ultrasound is usually used for physical or chemical alterations in the material properties [16].

Low-frequency and high-intensity ultrasound technology for enhancing several engineering and biotechnological processes has increased attention in the past two decades, especially driven by green process goals. One of these processes is extraction, notably for the recovery of bioactive compounds. Ultrasound-assisted extraction (UAE) consists of transferring acoustic waves to a medium composed of raw material and a liquid solvent. Current systems configurations are transducers coupled with a vessel and transducer probe immersed in extraction medium. Both configurations are available in batch modes. However, probe configuration has been reported to work also with the continuous solvent flow [13,17]. Although less economical and more challenging to operate, probe systems are commonly preferred than batch systems due to the higher ultrasonic intensity. The probe diameters for laboratory scales range from 2 mm (for volume up to 5 mL) to 25 mm (for volume up to 1 L) [18].

The UAE mechanism is described by acoustic cavitation, thermal, and mechanical effects. The application of ultrasonic waves causes expansion and compression cycles in the material. The expansion may create bubbles in the liquid solvent with internal negative pressure. The compression collapses such bubbles, promoting cavitation. These bubbles collapse near the cell walls of the raw material, producing cell disruption and resulting in solvent penetration that increases the mass transfer [16,19]. In addition to the cell disruption, other phenomena are produced, such as fragmentation, localized erosion, pore formation, increased absorption, and swelling index in the solid matrix of the raw material [13,18]. During the collapse, hot spots and extreme local conditions are produced, resulting in thermal effects; temperature may reach up to 5000 K, and pressure increase may be up to 10 MPa [18,20]. Moreover, shear forces and turbulence at the moment of collapse are produced, mechanically affecting the system [21].

The system design (as described above), extraction process parameters, electricity consumption, solvent type, solvent-to-feed ratio, and matrix particle size are the main factors that should be considered during the extraction process design. Regarding the system design, optimal vessel configuration and location of transducer-related elements should be considered to obtain the maximum energy transfer to the medium [21,22].

UAE process parameters are frequency, ultrasonic power or intensity, and amplitude. For obtaining the best cavitation effect, mainly frequency and intensity should be investigated; the high frequency suppresses the cycle of compression-expansion, making it more challenging to induce growth of cavitation bubbles due to the short period of the cycle. Therefore, low-frequency ultrasound is preferable for the extraction process [21,23]. There are few studies in the recovery of bioactive compounds varying the frequency. Most of the works deal at constant low-frequency (20–120 kHz), but 20 kHz is mainly employed [18]. Regarding the ultrasound power, the increase usually enhances the extraction yields [13]. The extraction of bioactive compounds is performed between 20–700 W. However, in some cases, the increase in ultrasound power to a very high level can decrease the effect on yield due to the increase in the number of bubbles formed; a high concentration of high bubbles volume leads to an inter-bubble collision, deformation, and nonspherical collapse, resulting in less impact between bubbles and raw material. Another consequence of high power is forming a layer of bubbles around the probe that hinders the transmission of the energy into the extraction medium and the degradation of bioactive molecules both due to thermosensitivity and molecular changes [18].

The solvent selection, as well as for other extraction methods, should consider the chemical characteristics of target compounds and solid matrix. Moreover, the solvent physical properties impact the cavitation intensity in UAE, i.e., it decreases with the increasing vapor pressure of solvents and surface tension [21,24]. Also, the ratio between solvent mass to feed mass (S/F) should be taken into account; low S/F may reduce the yield due to an insufficient amount of solvent to solubilize the available target compounds and, depending on the sample, due to the increase in the viscosity that reduces the cavitation [18]. In contrast, excessive S/F may reduce the extract concentration and increase the cost for solvent evaporation.

Temperature also has an interesting effect on UAE. The increase in temperature improves the target compounds disruption from the raw material, reduces the solvent viscosity, and decreases the surface tension between raw material and solvent. These factors enhance the solubilization and mass transfer of solute. The reduction in the solvent viscosity also favors cavitation. However, over a certain temperature point, an adverse effect may be observed, which means the medium properties favor the cavitation at a high level, resulting in the same effect of high powers [18,21,25].

Regarding time, it exhibits similar behavior of power and temperature; the increase in time increases the yields up to a certain point, and after, a low effect or reduction in the yield is observed [22]. It is worth mentioning that each extraction run has an overall extraction curve, which means the extraction rate slows down with increasing time since the diffusion drives the extraction after a particular time. The increase in the extraction rate by applying UAE is expected compared to other extraction techniques, especially during the first extraction periods, as a result of the aforementioned ultrasound effects; therefore, reducing the spend time to achieve the diffusional period. On the other hand, the reduction in the yield of target compounds by increasing the time of exposure to the ultrasound may be associated with degradation also due to thermosensitivity or molecular changes induced by the sonication. Additionally, to overcome such limitations, the exposure time may be reduced [13].

It is important to point out that although UAE has been widely employed for a wide range of raw materials, the ultrasound can be applied to enhance extraction processes in distinct ways. That means, it may be used as raw material pre-treatment for followed solid-phase extraction processes [16] or as an enhanced technique in the cases of hybridization extraction processes (see Section 2.3).

### 2.2. High-Pressure Technologies

High-pressure technologies have been extensively investigated in recent decades to obtain bioactive compounds from several sources, including plant and animal raw materials and biotransformation products [26]. Namely, supercritical fluids extraction (SFE), pressurized liquids extraction (PLE), and gas expanded liquids extraction (GXLE) are the main techniques. They have common advantages such as applying green solvents, higher extraction yields, selectivity, a high level of automation, extraction in dynamic mode, and feasible scheduling. In this section, we will go deeper into the fundamentals of each of these techniques.

#### 2.2.1. Supercritical Fluid Extraction (SFE)

SFE is a well-established technique both scientifically and industrially. A wide range of materials has already been submitted to SFE on a laboratory scale, mainly evaluating the effect of process variables on the extraction yield and concentration of target compounds in the extract. While on an industrial scale, this technique is used to obtain caffeine-free coffee, bitter compounds from hops, production of oils and spices from several plants, and nicotine from tobacco [27,28].

The potential of SFE is associated with the characteristics of the supercritical fluid. Above the critical temperature (Tc) and pressure (Pc), the fluid assumes densities of the magnitude of liquids and viscosities of gases, which improves its performance in the dissolution of compounds and the diffusion through the solid matrix of the raw materials [29]. The most used fluid is carbon dioxide (CO_2_), whose thermodynamic characteristics allow recycling by varying its temperature and pressure [20]. In addition to being recycled, CO_2_ is non-toxic, non-flammable, and can be obtained at a relatively low cost [30]. Additionally, CO_2_ at temperature and atmospheric pressure becomes gas, a characteristic that allows easy ventilation from the extract. Consequently, CO_2_ is easily removed from the extract by the pressure drop, leaving the extract utterly free of a solvent without additional energy expenditure [31]. Mild Tc and Pc (31 °C; 7.4 MPa) make it an excellent candidate for the extraction of low polarity compounds, especially those that are thermosensitive and susceptible to oxidation [32].

The main process variables that impact the yield and extract concentration are temperature, pressure, flow rate, time or S/F, solid particle size, raw material moisture, and capacity of the high-pressure reactor [33,34]. Notably, temperature and pressure have the greatest effect on the process as they impact the density of the solvent and the vapor pressure of the solutes. The minimum temperature and pressure limits are Tc and Pc, while the maximum limits do not exceed 100 °C (although most cases process up to 60 °C) and 45 MPa (although it is possible to operate at a very high pressure of about 100 MPa). The extraction mechanism can be driven by the density of the fluid or the vapor pressure of the target compound. In the first case, the increase in pressure and the decrease in temperature increase the density of the supercritical fluid and, therefore, increase the extraction yield. On the other hand, in processes governed by the vapor pressure of the solute, the increase in temperature favors its extraction [35]. It should be noted that the combination of temperature and pressure, as they affect the solubility of solutes, change the solvent’s tunability, i.e., make it more or less selective [36]. For instance, Viganó, Coutinho, Souza, Baroni, Godoy, Macedo and Martínez [37], by adjusting the pressure and temperature of the supercritical CO_2_, extracted three distinct fractions from passion fruit bagasse: tocopherol and tocotrienols-rich extract (60 °C; 17 MPa), fatty acids-rich extract (50 °C; 17 MPa), and a fraction more concentrated in β-carotene and β-cryptoxanthin than the first two fractions (60 °C; 26 MPa). This property can also be applied in separating the CO_2_ from the extract, where the fractionation of the extract is achieved by the pressure drop in different separators operating at different temperatures and pressures [38].

Overall extraction curves can be obtained by plotting the yield or the accumulated mass of extract versus the processing time or the S/F. A typical extraction curve has three well-defined regions associated with three mass transfer periods in the process. The first period corresponds to mass transfer by convection, in which the supercritical fluid solubilizes the easily accessible extract; this period is called the constant extraction rate period (CER). The second is the falling extraction rate period (FER) and is characterized by a decrease in easily accessible extract, and convection and diffusion govern the extraction. Finally, in the third period, called the diffusion-controlled rate period (DC), the mass transfer occurs by diffusion from the interior of the particles of the solid matrix to the surface of the particle [39].

The main limitation of supercritical CO_2_ is its low polarity; it is a good candidate for nonpolar compounds. However, it is unsuitable for most pharmaceutical and drug samples that are polar [20]. Low concentrations of cosolvent can be used to overcome this limitation, changing the polarity of the supercritical mixture and targeting more polar substances. Alternatively, PLE can be applied to obtain polar compounds like phenolic acids, flavonoids, stilbenes, and tannins, among others.

#### 2.2.2. Pressurized Liquid Extraction (PLE)

Pressurized liquid extraction emerged as a sample preparation technique combining high temperature and pressure with liquid solvents to achieve fast and efficient removal of the analyte from the solid matrix [40]. However, over the past two decades, given its advantages, it has been explored as a technique for obtaining compounds of industrial interest in the areas of food, drugs, nutraceuticals, and cosmetics, among others. Consequently, a vast amount of work has been published to study process variables and scale-up [41,42].

PLE can be found in the literature with other names such as pressurized solvent extraction, subcritical fluid extraction, accelerated solvent extraction, and enhanced solvent extraction [43], and for cases in which water is used as the solvent, pressurized hot water extraction, sub-critical water extraction, or superheated water extraction [44]. Despite this, the technique principle is always very similar and consists of raising the solvent’s temperature and pressure or mixture of solvents that pass through the extraction bed. The pressure has a strategic function as it allows the solvent to remain liquid even above its boiling temperature. Consequently, the solvent achieves some features important for the process: (i) high temperatures imply a reduction in viscosity and surface tension between the raw sample and solvent, consequently the ability of the solvent to permeate the pores of the solid matrix and the mass transfer are increased; (ii) the increase in temperature favors the breaking down target compound-matrix bonds and its diffusion to the matrix surface; (iii) high temperatures, generally above the boiling point and below the critical point, decrease the relative static permittivity, for instance, water at 25 °C and 0.1 MPa presents relative permittivity of 78.5, and at 350 °C and 17 MPa it decreases to 14.1 [27,43,44,45].

As a rule of thumb, the solvent choice impacts the yield and the selectivity of the PLE. The most used solvents are water and ethanol since they are recognized as safe. However, more recent research has pointed to novelties in the solvent options, namely the addition of modifying additives and the development of solvent gradients during extraction. The addition of modifiers aims to change the physicochemical properties of the solvent to improve some aspects during extraction. For example, weak acids such as citric acid are added to the solvent to better extract and preserve anthocyanins in the extract [46,47]. Also, the use of deep eutectic solvents and natural deep eutectic solvents in extraction techniques has grown in recent years [48]. Depending on the precursors used in the formulation and the composition of the solvent mixture, it may improve the extraction performance. Regarding the extraction methods that use solvent gradient, pumps able to make the selection and precise mixing of the solvents are required; therefore, a certain degree of automation is also required. However, there is the advantage of producing extract fractions concentrated in different compounds and with low contamination between them. For example, from complex raw materials in polyphenols, water can be used to extract compounds of high polarity such as phenolic acids initially, and then mixtures of water and ethanol and pure ethanol can be applied to obtain less polar compounds such as flavonoids [49,50].

The above-reported PLE features have several advantages over other extraction techniques. High mass transfer rates impact the time required to accomplish the extraction processes; consequently, the solvent amount is also reduced [40], and both time and solvent consumption impact the cost of manufacturing. Moreover, the extract leaves the extractor free of the solid matrix once the extraction bed works as a filter, and in general, the system has filtering elements to avoid pipeline clogging. Additionally, since the extract leaves the extraction vessel free of solid particles, it is very convenient to coupling online separation elements to concentrate the extract, for instance, solid-phase extraction elements [49]. However, PLE has some disadvantages, for instance, evaporating the solvent from the extract, which increases costs due to the time required and the expense of equipment and energy. High temperatures can be inconvenient for thermosensitive compounds [27] and favor undesirable compounds’ co-extraction [40]. Alternatively, solvent evaporation can be overcome by the specific design of an extract whose solvent has co-application. Interestingly, Strieder, Neves, Silva, and Meireles [51] used milk as a solvent to obtain a blue dye from genipap for application in food without the need to separate the solvent. Although this example did not apply PLE, it shows the importance of a specific design for applying the extract-solvent complex.

#### 2.2.3. Gas-Expanded Liquids (GXLs) Extraction

Gas-expanded liquids are liquids whose volume is increased by the addition of some compressed gas. In cases where CO_2_ is used, the technique is called CO_2_-expanded liquids (CXLs) [52]. In this way, at least two fluid phases or a single-phase exist (above the bubbled-point curve and below the critical point) [53]. In other words, GXLs demonstrate intermediate behavior between SFE and PLE for extraction of medium-polar compounds. The addition of compressed gas to a liquid solvent increases the mixture volume but does not necessarily decrease the density. Consequently, diffusivity increases by the solvent properties changing, i.e., interfacial tension and viscosity are decreased. Moreover, CO_2_ added to pressurized ethanol decreases the mixture’s polarity. Such behavior makes the CXLs good candidates for extraction and mobile phase in chromatography [52].

The advantages are associated with an increase in the yield with low consumption of organic solvents compared to classic solid-liquid extractions [53]. Moreover, mild pressures (3–8 MPa) allow reducing the energy consumption and the cost of manufacturing [54]. As the technique has behavior between SFE and PLE, it has potential application into the biorefinery context; for instance, SFE can be applied to treat some lipid-rich raw material, and before it is processed by PLE, it can be subjected to CXLs to recover medium-polar compounds [55].

Jessop and Subramaniam [56] classified the GXLs into different classes regarding their properties. Liquids that do not dissolve compressed liquid CO_2_ like water are defined as Class 1, and therefore properties are not significantly changed. Class 2 comprises liquids able to dissolve compressed CO_2_, e.g., methanol, hexane, and ethanol, resulting in changed properties. Furthermore, Class 3 includes liquids that moderately dissolve compressed CO_2_ like ionic liquids, polymers, and crude oils.

CXLs are still relatively unexplored as an extraction technique. Some examples are: obtaining highly polar natural pigments (crocin-1 and crocin-2) from *Gardenia jasminoides* Ellis fruit pulp using ethanol and water mixtures (50–80%, *v/v*) at 5–25 °C, 8–14 MPa, and sonication time (0–200 s) [57]; the extraction of gamma-linoleic acid from *Arthrospira platensis cyanobacteria* at 10–50% (*v/v*) ethanol, 40–80 °C, and 10-30 MPa [58]; and astaxanthin extraction from *Haematococcus pluvialis* microalgae at different ethanol contents of 50–70 wt.%, temperatures of 30–60 °C, at 7 MPa [59].

Regarding the instrumentation, the equipment is not very different from that traditionally used in SFE. Nevertheless, it is important to consider that a significant number of solvents can be employed in each extraction run; the system must consider pumping such solvents or mixtures. Likewise, in SFE and PLE, the system requires solvent and co-solvent pumps, heating systems, medium-high pressure vessels, valves, containers for solvent and extract, and instrumentations like manometers and thermocouples [55].

### 2.3. Low-Frequency Ultrasound Coupled with High-Pressure Technologies

Low-frequency ultrasound technology can be coupled with high-pressure extraction processes in different ways, namely: the raw material can be pretreated in ultrasound equipment separately from the high-pressure extraction equipment; it can be ultrasonically pretreated inside the extractor by coupling the ultrasound to the extraction vessel; and also for equipment that has ultrasound coupled with the extraction vessel, sonication can occur in pulses or while the high-pressure extraction process lasts. Also, there are different ways to couple the ultrasound to the extraction vessel; there are configurations that insert the extraction vessel into the ultrasonic bath and configurations that couple the ultrasound probe into the extraction vessel. This section will focus on the process fundamentals in which the probe configuration is used, and consequently, ultrasound can be applied in pulses or permanently during the extraction.

#### 2.3.1. Low-Frequency Ultrasound Coupled with SFE

Although SFE has several industrially established applications, extract production from some raw materials is not economically viable. Furthermore, in some cases, the yield and quality of the extract are comparable to those obtained by conventional methods. In the last decade, several works have been published, proposing the coupling of ultrasound with SFE to overcome such limiting aspects. In addition to improving these aspects, the coupling of ultrasound can enhance the extraction kinetics, especially at lower extraction temperatures when the vapor pressure of the solute limits solubility.

**Figure 2:**
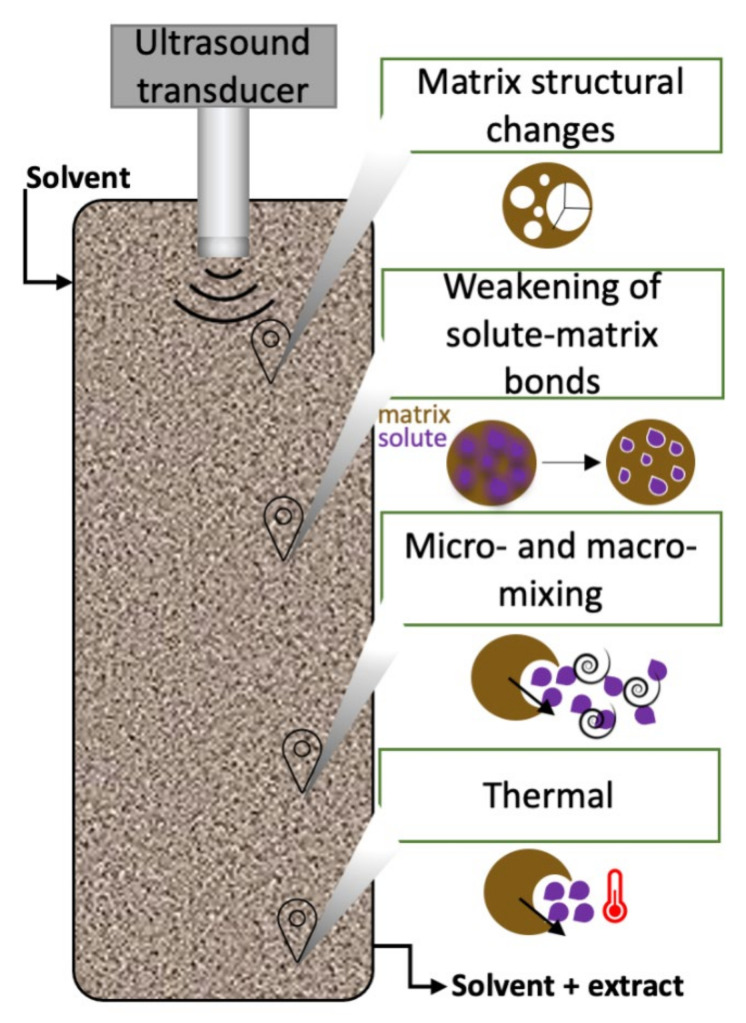
Extraction driving mechanisms of high-pressure and low-frequency hybrids systems.

However, the mechanisms involved in ultrasound coupled with SFE have only recently begun to be elucidated. The lack of phenomena explanation and difficulty in transposing the production scale still present limitations for scaling up the hybrid process. Faced with this, Dassoff and Li [28] proposed the description of the phenomenon through three mechanisms. The authors proposed that the mechanisms present in UAE are different from those of SFE coupled with an ultrasound since bubble cavitation rarely occurs due to the pressurization of the system and the lack of a phase boundary in the supercritical fluid. However, the literature shows an improvement in extraction kinetics when ultrasound is applied to SFE, which may be associated with other mechanisms, which we tentatively represent in Figure 2. The first is defined as physical cell damage due to local pressure difference, in which, due to local oscillations in pressure, enlargement of pores and damages on cell wall may occur, changing the solid surface area and matrix morphology. Another mechanism is the weakening of solute-matrix bonds favored by the application of an ultrasound; however, this mechanism lacks experimental proof. The third one described was the micro-mixing to facilitate solute movement and improve solvent accessibility; the ultrasound may accelerate the solute movement along the wavefront and promote vibrational friction, thus enhancing solute movement from the inner to the particle surface and the bulk solvent; furthermore, macro-mixing minimizes the concentration gradients into the extraction vessel due to macro-scale convective currents.

The coupled process parameters are very similar to regular SFE but with the addition of parameters from the ultrasound, like ultrasound power that is the most studied. However, it is worth mentioning that the ultrasound effect could depend on the vessel volume and the mass of raw material used; thus, it is advised to report the energy density (J/m^3^) or the specific energy (J/g). Special attention to the increase in temperature within the vessel must be given when different power conditions are offered, since different powers may result in different temperatures inside the extractor. Temperature exhibits a strong effect in SFE since it affects the solvent density and therefore impacts the extract solubility; also, temperature increases the solute vapor pressure. Thus, temperature variation changes the SFE solvating power. Based on that, we added a mechanism to those described by Dassoff and Li [28], i.e., the fourth mechanism (Figure 2), in which thermal effects are induced by the ultrasound application, raising the temperature inside the extractor. Thereby, Figure 2 refers to the driving mechanisms of high-pressure and low-frequency hybrids systems.

#### 2.3.2. Low-Frequency Ultrasound Coupled with PLE

PLE coupled to ultrasound, like SFE, has been used in recent years to improve PLE performance. Studies have shown a strong influence on the hybrid technique compared to regular PLE. For example, Viganó, Assis, Náthia-Neves, Santos, Meireles, Veggi, and Martínez [60] observed an increase in piceatannol yield of approximately 50% when PLE was coupled with ultrasound in the extraction from defatted passion fruit bagasse; however, the application of different nominal powers (240–640 W) did not affect the extraction yield. Similarly, Pereira, Zabot, Reyes, Iglesias, and Martínez [61] observed similar increase when the extraction was coupled with ultrasound (360 W/cm^2^) in obtaining total phenolics from passion fruit rinds.

Despite the strong influence of the hybrid technique, the few published works still do not effectively demonstrate the extraction action mechanism. Similar to SFE, the pressurization of the system can affect the development of bubbles, and consequently, the effects can be associated with mechanisms other than cavitation. Interestingly, Viganó, Assis, Náthia-Neves, Santos, Meireles, Veggi, and Martínez [60] investigated the temperature evolution throughout the extraction process and compared regular PLE with PLE coupled with ultrasound. Regular PLE was performed at 65 °C, and at the same conditions, ultrasound was coupled (240–640 W), resulting in the temperature increasing (75 °C); they also compared PLE coupled with ultrasound with the regular PLE at 75 °C. The authors concluded through chemical characterization and scanning electron microscopy analysis that the thermal effects of ultrasound caused improvement in the extraction kinetics of the hybrid process, corroborating with the thermal mechanism described in Section 2.3.1 and shown in Figure 2. On the other hand, Pereira, Zabot, Reyes, Iglesias, and Martínez [61] observed the effect of ultrasound intensity in obtaining total phenolics from passion fruit peel; at 240 W/cm^2^, the yield was significantly lower than at 360 W/cm^2^; however, 480 and 600 W/cm^2^ did not differ from each other and with 360 W/cm^2^. The authors concluded that the power offered to the system needs to reach a minimum value sufficient to overcome the hydrostatic pressure at the probe tip, which they identified as 360 W/cm^2^ for the adopted system.

These controversial observations may be produced by the different raw material characteristics, such as porosity, specific surface area, and particle size, making each raw material respond differently to the application of ultrasound in PLE. In fact, the size of solid particles has been shown to have an effect associated with ultrasound. Sumere, Souza, Santos, Bezerra, Cunha, Martinez, and Rostagno [62] evaluated different powers in two different particle sizes and found that, regardless of power, the total phenolics yield was significantly affected by particle size. Smaller particles (0.68 mm) interestingly showed higher yields than larger particles (1.05 mm). This behavior can be associated with some of the mechanisms described in SFE coupled with ultrasound, such as micro-mixing, which through vibrations, promotes greater diffusion of the solvent into the particle and from the particle’s interior to the surface, intensifying the mass transfer.

In order to favor the formation and collapse of bubbles during the PLE coupled with ultrasound to promote cavitation effects that are usually mitigated by solvent pressurization, Santos, Souza, Sumere, da Silva, Cunha, Bezerra, and Rostagno [63] proposed the application of N_2_ to expand the PLE solvent, previously called gas-expanded liquid extraction. The authors identified both ultrasound power and expansion gas initial pressure as factors that significantly affected the extraction yield of target compounds. The use of expansion gas (N_2_ initial pressure of 0.5 MPa) without ultrasound increased the total phenolic compounds yield by approximately 10%. However, when the amount of gas in the liquid was increased (N_2_ initial pressure of 1.5 MPa), the yield drastically reduced (37.4%), indicating the existence of limits to explore this variable. Interestingly, this reduction in yield was not observed when expansion (N_2_ initial pressure of 0.5 MPa) was associated with ultrasound (400–600 W), indicating the possible influence of liquid expansion on the cavitation process by facilitating the initial stage of bubble formation as well as bubble size, number, and implosion. The acoustic cavitation phenomenon contributes to the disruption of intermolecular bonds and solvent accessibility to the sample, improving yield. Therefore, the results and findings obtained by Santos, Souza, Sumere, da Silva, Cunha, Bezerra, and Rostagno [63] corroborate the mechanism described by Dassoff and Li [28] to explain the mechanisms involved in the coupling high-pressure extraction techniques with low-frequency ultrasound, especially showing that at high-pressure, solvents may lessen some of the ultrasound effects that are seen in low-pressure systems. In the face of the issues described in this section, new investigations focused on elucidating and demonstrating the extraction mechanisms in hybrid high-pressure techniques with low-frequency ultrasound being required.

## 3. Innovative Processes for the Extraction of Phytochemical Compounds

Extraction is a unit operation that plays a remarkable role in areas such as food, agriculture, chemical, pharmaceutical, and cosmetics, among others. The extraction of oils from oleaginous seeds is one of the most common techniques known worldwide. Even though, in the last years, the extraction of specific compounds has been the focus of many companies to comply with the consumers’ rapid-changing demand. The extraction of terpenoids, carotenoids, polyunsaturated fatty acids, proteins, sterols, phenolic acids, and alkaloids, among others, is increasing everywhere, especially in developing regions. Vegetal, microalgae, and fungal biomasses are the main sources used for the extraction of target compounds. Active value-added substances have an important role because they can either satisfy the consumers’ demand or provide profitable trading. Consequently, technologies are needed to recover the main active substances targeted in each area.

Several emerging technologies have been employed to recover phytochemical compounds from biomasses inside a biorefinery approach or even aiming for bioactive compounds extraction from by-products or residues. Innovative techniques such as low-frequency ultrasound [64,65], supercritical CO_2_ [34,66], pressurized liquids [47,67], high-pressure processing [68,69], pulsed electric fields [70,71], and others, allow the recovery of bioactive compounds using non-thermal treatments and green solvents. In this way, the extracts obtained by these techniques are promising in technological applications for enhancing food quality and health attributes since they are free of toxic solvents.

### 3.1. Low-Frequency Ultrasound as a Single Operation

Ultrasound extraction is a unit operation that uses acoustic waves to reach the target compounds by traveling through a solvent. In most cases, water and ethyl alcohol are the solvents used. Also, methyl alcohol, hexane, dichloromethane, propyl alcohol, acetone, and ethyl acetate, among others, can be used, depending on the polarity of the active compound and the application area. Indeed, non-GRAS (Generally Recognized As Safe) solvents have limited use because they can restrict the products where the compounds can be incorporated.

The studies and operation lines use ranges of frequency to cause different cavitation bubbles and acoustic power to intensify the liquid system’s turbulence. When cavitation bubbles reach the solid surface of the biomass, they burst and cause shockwave-induced damage to the cell wall. The implosion of the cavitation bubbles results in liquid jets of ultra-high velocity. Consequently, the bioactive compound is released from the core-cell and it is transferred to the solvent solution. There is a combination of process parameters that maximize the extraction yield, such as mainly temperature, frequency, processing time, and S/F ratio. Overall, the energy is high and the frequency is low, ranging between 16–100 kHz. Generally, most of the studies and applications report frequencies from 20–40 kHz.

Frequently, ultrasound extraction is reported to have advantageous results because the cavitation phenomenon increases the mass transfer and, consequently, the extraction yields. This is the case of extraction of polysaccharides from Crataegus pinnatifida Bunge using ultrasound with water as solvent at 60 °C for 60 min, whose yield (7.47 ± 0.05 wt%) was approximately 27% higher than the yield obtained with traditional hot water extraction at 90 °C for 120 min (5.88 ± 0.19 wt%) [72]. Likewise, the use of ultrasound to obtain an extract from baobab (Adansonia digitata) seeds resulted in a significantly higher total flavonoids content (1648.18 ± 10.75 mg rutin equivalent/100 g dry raw material) compared with maceration (1261.29 ± 2.39 rutin equivalent/100 g dry raw material) and heat-assisted extraction (1156.14 ± 2.34 mg rutin equivalent /100 g dry raw material). Also, the total phenolics content (418.01 ± 9.49 mg gallic acid equivalent/100 g dry raw material) was approximately 17% and 20% higher than the contents in extracts obtained by maceration and heat-assisted extraction, respectively [73].

In addition to better results in most of the cases, ultrasound extraction is performed in simple equipment. In sonochemistry, this technology needs a generator that generates ultrasonic waves to be dissipated through the solvent using an ultrasonic probe made of titanium or titanium alloys, for example. Ultrasonic baths or cup horns can be used as well. Ultrasonic baths generate intensities from 1 to 5 W/cm^2^. However, the distribution of the waves is not homogeneous and part of the energy is also dissipated through the fluid that fills the reservoir. Otherwise, ultrasonic probes generate intensities from 50 to 750 W/cm^2^. They can provide more energetic conditions because they can be directly inserted into the extraction mixture, which improves energy transfer. It is important to emphasize that both these intensities refer to the nominal powers. The propagation of ultrasonic waves causes the implosion of bubbles, thus causing intense local turbulence, particle collisions, and high local pressure. For larger applications, continuous procedures are preferred to batch systems. Ultrasonic baths or probes are used in flow systems where fine particles of biomass dispersed in a liquid solution flow through. The sonication can be in direct mode (direct contact) or indirect mode (no contact between a dipped probe and the mixture). A probe or horn system is preferable because the intensity is delivered on a small surface (more ultrasound intensity, W/cm^2^) compared to the bath. Table 1 lists some of the main findings reached using ultrasound as a single operation for the extraction of target compounds.

Regarding patents, many inventions claim specific processes. This is the case of one invention that presents an ultrasound-assisted process for efficient and cost-effective production of commercially value-added products with zero waste from both fermented and unfermented types of cocoa beans [82]. It was achieved by aqueous, hydro-acetone (minimum 20% water, *v/v*), and hydro-alcoholic (minimum 20% water, *v/v*) extractions mediated by ultrasonication-assisted enzymatic treatment at a temperature ranging from 50 °C to 60 °C. Sonication is applied as intermittent pulses, such as 1 min to 30 min, and proteases, amylases, and amyloglucosidases are used combined with ultrasound to extract soluble and de-bittered cocoa dietary fiber rich in proteins. The liquid extract containing cocoa extractives (referred to as miscella) and the solvent are submitted to approximately 60–80 °C for evaporating the solvent. The evaporation is stopped when a total dissolved solid level of approximately 15–20% (*w/v*) is achieved. Thereafter, it is mixed with sugar syrups and food-grade emulsifiers and is further concentrated for 3–6 h at 100–120 °C until having a free-flowing brownish liquid. The residual solvent level is evaluated and, if it is less than 20 ppm, the product is suitable for food and beverage applications, especially with characteristics like chocolate aroma and taste [82].

Another patent describes an apparatus and a method for extracting bioactive compounds from natural sources using a counter-flow extractor (inclined casing containing a helical conveyor screw having a plurality of blades) assisted by a sound transduction system. The use of ultrasound improves the extraction yield at a given temperature, allowing for low S/F ratios compared to a normal continuous extractor (without ultrasound). The authors claim that the invention consumes less energy (acoustic power), operates at lower temperatures, uses less amount of solvents, and minimizes the area and cross-section necessary for the application of ultrasound. Charts with positive results of a study carried for determining the influence of ultrasound on saponin extract are presented [83].

### 3.2. Low-Frequency Ultrasound Coupled with High-Pressure Technologies

One of the advantages of using ultrasound technology for extractions is its possibility of coupling with other technologies. One integration commonly done is with high-pressure technology, in which ultrasound assists the recovery of a broader range of compounds from specific biomass.

In the food engineering area, malagueta pepper (*Capsicum frutescens* L.) was processed by supercritical CO_2_ extraction assisted by ultrasound [84]. The association of ultrasound with the SFE increased up to 77% of the global yield of extract, reaching 97 ± 10 mg/g dry pepper. Capsaicin, dihydrocapsaicin, nordihydrocapsai-cin, and homodihydrocapsaicin were identified in the extracts [84]. Capsaicin is a target compound because it presents anti-inflammatory, antioxidant, antitumor, and antioxidant activities [85]. According to Santos, Aguiar, Barbero, Rezende, and Martínez [84], ultrasonic waves integrated with supercritical CO_2_ did not influence significantly the phenolic and capsaicinoids contents in the extracts. This is a positive finding because the ultrasound enhanced the extraction yield and, consequently, the capsaicinoids yield as well.

Another example is reported with passion fruit bagasse (*Passiflora edulis* sp.). PLE assisted by ultrasound was used to intensify the extraction of phenolic compounds, resulting in 60% more total phenolics (approximately 35 mg gallic acid equivalent per g dry bagasse) and piceatannol (approximately 6.5 mg/g dry bagasse) if compared to single pressurized liquid extraction at the same condition of pressure and temperature [60]. Overall, phenolics are bioactive compounds applied worldwide as active ingredients in many formulations. Therefore, researches like that one aforementioned are useful to show the potential of ultrasound waves to increase the mass transfer of specific and value-added solutes. Table 2 lists some of the main findings reached using ultrasound coupled to high-pressure technology for the extraction of target compounds.

In Table 2, most of the parameters used in the extractions are scalable, such as pressure, temperature, frequency, time, and S/F ratio. However, the ultrasound power (W) is associated with the mass and volume used in the extractions. Therefore, the energetic density of sonication (J/m^3^ or W.s/m^3^) is preferred to be used because it means the power transmitted to a known volume of mixture for a known time, which could be used in scale-up designs and projects. Another possibility is using the power density (W/m^3^). For example, according to Donadone, Giombelli, Silva, Stevanato, Silva, and Barros [93], the lowest extraction of phenolic compounds from the stem portion of peach palm (*Bactris gasipaes*) was achieved for the highest levels of ultrasound power density (25.42 W/L). One inference is that a high-power density can cause the degradation of phenolics because the collapse of the bubbles generated by the cavitation is extremely intense, thus reaching high temperatures. Otherwise, according to González-Centeno, Knoerzer, Sabarez, Simal, Rosselló, and Femenia [94], power densities of 50 W/L, 100 W/L, and 150 W/L were evaluated on the extraction of total flavonols from grape pomace (*Vitis vinifera* L.). The authors reported an increased recovery of total flavonols with increasing power density, with the increment more remarkable at the highest ultrasound frequencies (100–120 kHz), thus presenting from 1.1 to 1.6 mg quercetin/100 g fresh weight. Indeed, in most cases, the parameters have an interaction with each other. Solutes that need extreme extraction conditions are favored with high ultrasound energetic densities. Regarding the power density, the physicochemical nature of the fresh biomass, the target compounds, and the frequency can influence the expected results. This is why the scientific knowledge in this area is extremely dependent on extensive experimental assessments to have a way forward.

Considering a brief patent survey, some inventions can be highlighted. One invention refers to a high energy ultrasound extraction method and apparatus, which claims a process and equipment that uses low-frequency ultrasound (16–100 kHz) for extractions. A wide range of compounds can be extracted, which includes, but is not limited to, flavorings, colorings, and nutraceutical substances existing within the raw material. The extent of the biomass load and type of structure of the organic substrate determine the type of sonotrode design to have an enhanced efficiency. The apparatus allows the use of low-frequency ultrasound combined with supercritical fluids, such as CO_2_, for the extraction of bioactive compounds. The invention presents extractable substances from oak materials within the range of 1–5 wt% [95].

Another novel invention claims an integrated system of analyses to determine chemical compounds using ultrasound and high-pressure technology. The system is formed by an extraction cell, a solid-phase extraction column, an ultrasound generator, a probe, and a chromatographic apparatus. The ultrasound-assisted extraction and supercritical fluid or pressurized liquid extraction are used to extract analytes from different samples and to quantify them by chromatography through a process integration approach. Examples of the viability of the system were presented with caffeine and phenolic compounds from coffee, and phenolic compounds from pomegranate [96].

## 4. Low-Frequency Ultrasound in the Industrial Scale

Regarding the worldwide application of this technology, many companies are specialized in the design and manufacturing of high-power ultrasonic equipment for different purposes or in the extraction procedure. Overall, some companies were randomly selected and cited herein (non-exhaustive list). The Hielscher Ultrasonics GmbH [97] manufactures ultrasonic devices for mixing, dispersing, particle size reduction, extraction, and chemical reactions. Considering the extraction operation, the devices comprise sizes from laboratory to industrial scales. For the laboratory, the devices comprise powers from 50 W to 400 W, frequencies from 24 kHz to 30 kHz, and volumes from 0.01 mL to 2000 mL. The types are stand-alone, handheld, or stand-mounted. For industrial, the devices comprise nominal powers from 500 W to 16,000 W, frequencies from 18 kHz to 20 kHz, and flow rates of the extractable mixture from 0.25 L/min to 15 L/min. Also, they can be configured to operate in parallel mode. For example, a 4 × 16 kW system has the capacity for an extraction performed with a flow rate from 1 m^3^/h to 12 m^3^/h.

The Reus [98] company also develops large-scale ultrasound extraction devices. Specific needs of other companies can be solved and supported by the creation of integrated plant extraction chains dedicated to phytotherapy; the pharmaceutical industry; and the production of vermouth, flavoring oil and vinegar, cognac, gin, and whisky, among others. The company has been present in the field of ultrasounds since the 1970s. It is specialized in the industrial manufacturing of specific and high-quality generators and transducers. The Sonics & Materials [99] is a USA company that designs and manufactures devices for ultrasound extraction. An example is the VCX 2500 equipment, which is a 2500 W processor with an air-cooled converter. It is capable of processing up to 50 L on a batch basis.

The Duas Rodas Flavors & Botanicals [100] uses 10 different methods and technologies to extract the active components from plants. One of the technologies is the ultrasound extraction, which they consider a method of process intensification that allows obtaining high extraction rates in lower processing times. The company produces natural extracts and ingredients that are on the rise in the global food and beverage market, which is aligned with the growing trend of consumers for naturalness, well-being, and health.

G. Mariani & C. Spa [101] agree with the worldwide technological developments and, therefore, created a production unit for extracts and infusions applying the ultrasound technology. Aromatic compounds from spices are extracted by this innovative technology with a reduction in the number of extraction cycles and, consequently, with the optimization of the processing time. They can select the most appropriate technique, based on the characteristics of aromatic herbs and their production requirements, and produce the widest range of extracts, infusions, and distillates.

Otherwise, to date, there is no ultrasound coupled to high-pressure technologies on the industrial scale or even companies that produce these devices [102]. However, this is not a new field of research that remains unexplored as discussed in this review. According to Dias, Aguiar and Rostagno [103], the scale-up of a high-pressure system assisted by ultrasound is still challenging for the companies that aim to expand their production from lab- to industrial scale.

## 5. Challenges and Future Perspectives

One of the challenges is related to the stability of thermolabile compounds. High ultrasound energetic densities, that is, high powers for long times applied to a small volume, can cause degradation of compounds of interest. Also, high temperatures and pressures reached inside the cavitation bubbles can reduce partially or totally the bioactivity of solutes. Therefore, ultrasound technology should be selected to process spices, herbs, vegetal wastes, and algae and fungal biomasses that have no extremely volatile target substances. Low energetic densities can be a solution to such situations. On the other hand, the action mechanisms of an ultrasound hybridized with high-pressure techniques still needs more clarification, comprising an opening field of research.

In some countries, another challenge is the lack of regulatory approval of products processed by ultrasound. The technology is not too recent, but the application is still not traditional. Therefore, some limitations exist, which can delay a wider implementation in specific regions. The necessity of fine particles is also one of the challenges because large particles can cause high resistance to mass transfer. Even though the ultrasound is a powerful process that ruptures hard particles, there is a need for high energetic densities (more power for longer times) when the particles are big. Then, high investment costs are needed to purchase stronger equipment.

Separation and purification are listed as a challenge because the extracts, after sonication, are dissolved in a solvent. Consequently, a further operation, such as evaporation, freeze-drying, or vacuum-drying, is needed to separate the solid or liquid extracts from the solvent. This cannot be generalized because the extract can be recovered at the end of the process without the solvent (if no modifier is used) when ultrasound-assisted extraction is coupled with supercritical CO_2_ extraction. The necessity of separation, as aforementioned, cannot be a rule because some aqueous or alcoholic extracts can be used in a dissolved mixture, thus facilitating the application. Once low volumes of solvents are used in some cases (low S/F ratios), ultrasound technology can produce green extracts in a certain concentrate form that can be used as they are. Regarding purification, this challenge is not specific for ultrasound technology, but all extraction methods. Even though ultrasound extraction can be selective, when a substance or class of substances is needed with high purity, a further operation, such as adsorption or membrane filtration, is needed to satisfy this purpose.

Despite the challenges, ultrasound presents promising future perspectives in process engineering to keep increasingly transferring knowledge into technology for commercial development. Once it uses physical and chemical phenomena instead of a biological phenomenon, the technology is easily scalable, and the data are reproducible. In this novel bioeconomy, ultrasound extraction systems are environmentally friendly. One future outlook is the expansion of the combination of ultrasound with high-pressure technology.

Indeed, based on a systematic search of new systems developed to date, ultrasound is a cutting-edge technology because operations such as emulsification, pasteurization, fermentation, enzymatic reaction, and extraction are beneficiated through this technology. In summary, ultrasound extraction provides higher yields, allows rapid batches, enables using green solvents, has simplicity, can preserve extract materials under a concentrated form, and is safe to run.

## Figures and Tables

**Table 1:** Extraction of selected target compounds from different biomasses using ultrasound as a single operation.

Target Compounds	Yield	Biomass	Temperature (°C)	Frequency (kHz)	Solvent	S/F (mL/g)	Processing Time (min)	Ref.
Flavonoids	14.4 mg/g oleoresin	*Cinnamomum burmannii*	30	80	Water	10	60	[74]
Flavonoids	29.8 mg/g oleoresin	*Cinnamomum burmannii*	30	80	Ethyl alcohol	10	60	[74]
Total soluble phenols	155 mg/g leaves	*Artocarpus heterophyllus* Lam.	10	42	Ethyl alcohol: water (80:20, *v/v*)	10	30	[75]
Triterpenoids	14.3 mg/g powder	*Ganoderma lucidum* spore powder	60	-	Ethyl alcohol: water (95:5, *v/v*)	50	10	[76]
Exopoly-saccharides	46.7 mg/g fungal mycelium	*Purpureocillium lilacinum* and *Aspergillus niger*	-	-	Water	-	10	[77]
Fatty acids	195 mg/g fungal biomass	*Mortierella isabellina*	10	24	Chloroform: methanol: water (2:1:0.8, *v/v/v*)	40	30	[78]
Fatty acids	625 mg/g seeds	*Cucurbita pepo*	-	20	Hexane	10	5	[79]
Terpenoids	210 mg/g extract	*Mentha piperita* L.	50	40	Methylene chloride	10	40	[80]
Carotenoids	2.2 ± 0.1 mg/L algae extract	*Ulva flexuosa*	40	-	Ethyl alcohol: water (7:3, *v/v*)	25	60	[81]

S/F refers to solvent mass to feed mass ratio.

**Table 2:** Extraction of selected target compounds from different biomasses using ultrasound coupled to high-pressure technology.

Target Compounds	Yield	Biomass	Integrated Technology	Temperature (°C)	Frequency (kHz)	Nominal Power (W)	Solvent	S/F (g/g)	Processing Time (min)	Ref.
Phenolic compounds	43.3 ± 0.8 mg GAE/g dry peels	*Punica granatum* L.	Pressurized liquid extraction—10 MPa	70	19	400	Water	-	10	[62]
Phenolic compounds	6.5 mg GAE/g dry leaves	*Origanum vulgare* L.	Supercritical fluid extraction—35 MPa	35	30	60	CO_2_: ethyl alcohol (97.7:2.3, *w/w*)	5.5	60	[86]
Capsaicinoids	1.5 ± 0.3 mg/g dry fruits	*Capsicum baccatum* L. var. *pendulum*	Supercritical fluid extraction—25 MPa	40	-	200	CO_2_	484	40	[87]
Luteolin	0.06 mg/g dry leaves	*Perilla frutescens* L.	Liquid CO_2_ extraction—10 MPa	25	20	188	CO_2_: ethyl alcohol (84.2:15.8, *w/w*)	-	2.1	[88]
Cucurbitacin	7.34 ± 0.05 mg/g dry seeds	*Iberis amara*	Supercritical fluid extraction—25 MPa	55	40	200	CO_2_: ethyl alcohol (88:12, *w/w*)	30	60	[89]
Lipophilic molecules	9 ± 1 mg/g dry straw	*Saccharum officinarum* L.	Supercritical fluid extraction—25 MPa	60	-	800	CO_2_	126	120	[90]
Fatty acids	455 mg/g oil (39.4 mg oil/g fungal biomass)	*Nigrospora* sp.	Supercritical fluid extraction—25 MPa	80	40	132	CO_2_: ethyl alcohol (50:50, *w/w*)	113	85	[91]
Lutein	1.2 mg/g algae biomass	*Chlorella pyrenoidosa*	Pressurized fluid extraction—25 MPa	24	20	1000	CO_2_: ethyl alcohol	-	240	[92]

S/F refers to solvent mass to feed mass ratio. GAE refers to gallic acid equivalent.
